# Covariate balance-related propensity score weighting in estimating overall hazard ratio with distributed survival data

**DOI:** 10.1186/s12874-023-02055-8

**Published:** 2023-10-13

**Authors:** Chen Huang, Kecheng Wei, Ce Wang, Yongfu Yu, Guoyou Qin

**Affiliations:** 1 Department of Biostatistics, School of Public Health, Fudan University, Shanghai, China; 2 Shanghai Institute of Infectious Disease and Biosecurity, Shanghai, China; 3 Key Laboratory of Public Health Safety of Ministry of Education, Key Laboratory for Health Technology Assessment, National Commission of Health, Fudan University, Shanghai, China

**Keywords:** Cox model, Distributed data networks, Privacy protection, Propensity score weighting, Covariate balance

## Abstract

**Background:**

When data is distributed across multiple sites, sharing information at the individual level among sites may be difficult. In these multi-site studies, propensity score model can be fitted with data within each site or data from all sites when using inverse probability-weighted Cox regression to estimate overall hazard ratio. However, when there is unknown heterogeneity of covariates in different sites, either approach may lead to potential bias or reduced efficiency. In this study, we proposed a method to estimate propensity score based on covariate balance-related criterion and estimate the overall hazard ratio while overcoming data sharing constraints across sites.

**Methods:**

The proposed propensity score was generated by choosing between global and local propensity score based on covariate balance-related criterion, combining the global propensity score fitted in the entire population and the local propensity score fitted within each site. We used this proposed propensity score to estimate overall hazard ratio of distributed survival data with multiple sites, while requiring only the summary-level information across sites. We conducted simulation studies to evaluate the performance of the proposed method. Besides, we applied the proposed method to real-world data to examine the effect of radiation therapy on time to death among breast cancer patients.

**Results:**

The simulation studies showed that the proposed method improved the performance in estimating overall hazard ratio comparing with global and local propensity score method, regardless of the number of sites and sample size in each site. Similar results were observed under both homogeneous and heterogeneous settings. Besides, the proposed method yielded identical results to the pooled individual-level data analysis. The real-world data analysis indicated that the proposed method was more likely to find a significant effect of radiation therapy on mortality compared to the global propensity score method and local propensity score method.

**Conclusions:**

The proposed covariate balance-related propensity score in multi-site distributed survival data outperformed the global propensity score estimated using data from the entire population or the local propensity score estimated within each site in estimating the overall hazard ratio. The proposed approach can be performed without individual-level data transfer between sites and would yield the same results as the corresponding pooled individual-level data analysis.

**Supplementary Information:**

The online version contains supplementary material available at 10.1186/s12874-023-02055-8.

## Background

The growth of large multi-site medical datasets is accelerating with the development of big data and advances in data collection and storage. If data from multiple sources can be combined, the study power and generalizability can be improved, and multi-site research collaboration can also be carried out. However, in research of data from multiple sites, it is generally challenging to share information at the individual level among sites due to privacy, network security, and transmission speed [1]. Therefore, it is necessary to develop statistical methods that only require summary-level information to provide personal privacy protection while analyzing data from multiple sites.

In biomedical research, a common outcome of interest is the time-to-event endpoint, which focuses on whether or not an event occurred and when that event occurred. Cox proportional model is a popular semi-parametric approach to describe the relationship between the time-to-event endpoints and a set of covariates by estimating the hazard ratios [2]. In multi-site, distributed data, Lu et al. and Vilk et al. developed distributed Cox model based on iterative methods, which required iterative data sets to be transferred multiple times between the analysis center and each site [3, 4]. Li et al. proposed a method for distributed Cox regression that did not need multiple iterative file transfers among sites, but used the summary-level statistical data received from each site to find the solution of parameters based on the iterative method in the analysis center [5].

In observational studies, the inverse probability weighted (IPW) Cox regression model can be used to estimate the overall hazard ratio while adjusting for measured confounders through weighting [6]. Propensity score is the probability of treatment assignment conditional on the covariates and the IPW method assigns weight as the inverse of the probability of receiving the observed treatment to each individual [7–9]. In multi-site, distributed studies, considering propensity score weighting, Yoshida et al. compared three methods of sharing aggregate-level information to assess the performance of estimating hazard ratio from cox models in simulated distributed data networks [10]. The estimated results were comparable to the pooled individual-level data analysis. Shu et al. estimated the hazard ratio in multi-site study based on the IPW Cox model with summary-level information and provided theoretical justification [11]. Most multi-site studies obtained estimation based on local propensity score and local weight which fit propensity score models using data within each site. The local propensity score considered the possible heterogeneity of each site, while the sample size used to fit models was reduced. Alternatively, a global propensity score model can also be fitted using data from all sites based on distributed logistic regression, and the estimated treatment effect will be equivalent to a weighted pooled individual-level analysis [12]. However, when there is unknown heterogeneity of covariates in different sites, either global or local propensity score to estimate the overall treatment effect may result in potential bias or lower efficiency.

In this article, we propose a new method that uses only the summary-level statistics from each site to estimate the overall hazard ratio based on the new proposed propensity score in distributed survival data. The proposed propensity score is generated by choosing between global and local propensity score based on criteria to better control confounding bias and improve estimation efficiency. Our proposed propensity score is motivated by Dong et al. who proposed the subgroup balancing propensity score to estimate the subgroup treatment effect, which combined the global and local propensity score estimation to ensure covariate balance and control variance inflation [13].

The rest of the article is organized as follows. In Sect. "Data transfer from each site k to the analysis center: each site transmits distinct observed event times for site k,, to the analysis center." in methods, we present the weighted estimation of overall hazard ratio through IPW Cox model. In Sect. 2 in methods, we present the proposed method to estimate the propensity score, and provide respective algorithms using summary-level information to obtain the proposed propensity score. In Sect. 3 in methods, we present the methods of solving the estimating equations to estimate the overall hazard ratio based on the proposed propensity score. In simulations section, we present the simulation results demonstrating the performance of the proposed method and compare that to the global or local propensity score method and pooled individual-level data analysis. In application section, we give a real-world data application for illustration. At the end of the article, we conclude with some discussion.

## Methods

### Weighted estimation of the overall hazard ratio

Let ${\varvec{X}}$ be a vector of measured confounders, $A$ be a binary treatment variable ($A=1$ if treated and $A=0$ if untreated). ${T}^{*}$ is the true survival time, *C* is the censoring time which assumed to be independent of ${T}^{*}$ given $X$. Due to censoring, we observe $T=\mathrm{min}\left({T}^{*},C\right)$ and $\delta =I({T}^{*}\le C)$.$I(\bullet )$ is the indicator function. Suppose we observe *n* independent sample $\left\{{A}_{i},{T}_{i}{,{\varvec{X}}}_{i}{,\delta }_{i}\right\}$, $i=1,\dots ,n$, from *K* data-contributing sites. Let ${\Omega }_{k}=\{i:i\mathrm{ in site }k,\mathrm{ for }i=1,\dots ,n\}$ be the index set for individuals belonging to the ${k}^{th}$ sites with size ${n}_{k}$ and ${G}_{i}=k$ if individual $i$ belongs to the ${k}^{th}$ site, where $k=1,\dots ,K$.

Suppose we have $d$ distinct observed event times across all sites where${\mathrm{T}}_{1}^{D}<{\mathrm{T}}_{2}^{D}<\dots <{\mathrm{T}}_{d}^{D}$. For$j=1,\dots ,d$, let ${D}_{j}$ be the set of individuals who have the observed event time of${\mathrm{T}}_{j}^{D}$,${D}_{j}=\{i:{T}_{i}={\mathrm{T}}_{j}^{D},{\delta }_{i}=1, i=1,\dots ,n \}$, and let ${R}_{j}$ be the risk set for individuals who are at risk at time${\mathrm{T}}_{j}^{D}$,${\mathrm{R}}_{j}=\{i:{T}_{i}\ge {\mathrm{T}}_{j}^{D}, i=1,\dots ,n \}$. Also, let ${R}_{j}(k)$ be the risk set for individuals who are at risk at time ${\mathrm{T}}_{j}^{D}$ in site$k$,${R}_{j}(k)=\{l:{T}_{l}\ge {\mathrm{T}}_{j}^{D}, l\in {\Omega }_{k}\mathrm{ for }l=1,\dots ,n\}$. Similarly, within site *k*, there are $d(k)$ distinct observed event times${\mathrm{T}}_{k,1}^{D}<{\mathrm{T}}_{k,2}^{D}<\dots <{\mathrm{T}}_{k,d(k)}^{D}$. For$j=1,\dots ,d(k)$, let ${D}_{k,j}({k}{\prime})$ be the set of individuals who have the observed event time of ${\mathrm{T}}_{k,j}^{D}$ in site${k}{\prime}$, ${D}_{k,j}({k}{\prime})=\{l:{T}_{l}={\mathrm{T}}_{k,j}^{D},{\delta }_{l}=1, l\in {\Omega }_{{k}{\prime}}\mathrm{ for }l=1,\dots ,n \}$ and let ${\mathrm{R}}_{k,j}({k}{\prime})$ be the risk set for individuals who are at risk at time ${\mathrm{T}}_{k,j}^{D}$ in site${k}{\prime}$*,*${\mathrm{R}}_{k,j}({k}{\prime})=\{l:{T}_{l}\ge {\mathrm{T}}_{k,j}^{D}, l\in {\Omega }_{{k}{\prime}}\mathrm{ for }l=1,\dots ,n \}$, where${k}{\prime}=1,\dots ,K$.

In this article, we focus on estimating the overall hazard ratio, $\mathrm{exp}(\theta )$, between treatment and control groups in the entire population:

$$\lambda \left(t\right)={\lambda }_{0}(t)\mathrm{exp}(\theta A)$$

where ${\lambda }_{0}(t)$ is the baseline hazard function.

IPW Cox regression model is commonly used to estimate hazard ratio. Based on the propensity score $e=P(A=1|{\varvec{X}})$, the inverse probability weight is $w=\frac{A}{e}+\frac{1-A}{1-e}$. We assume that the hazard ratio to be common across* K* data-contributing sites and all sites have a common baseline hazard ${\lambda }_{0}(t)$. The weighted partial likelihood score function for the common log hazard ratio $\theta$ is [14],

1 $$\sum_{j=1}^{d}\sum_{i\epsilon {D}_{j}}{w}_{i}\left\{{A}_{i}-\frac{\sum_{l\epsilon {R}_{j}}{w}_{l}\mathrm{exp}({\theta A}_{l}){A}_{l}}{\sum_{l\epsilon {R}_{j}}{w}_{l}\mathrm{exp}(\theta {A}_{l})}\right\}=0$$

The estimate of the log hazard ratio $\widehat{\theta }$ can be obtained by solving Eq. (1).

### Proposed propensity score weighting method for estimating the overall hazard ratio

We propose a new method to estimate the overall hazard ratio based on our proposed propensity score weight, which does not require individual-level data sharing among sites. Specifically, we first estimate the global propensity score for the entire population by distributed logistic regression and generate a global weight for each individual. Second, we fit logistic regression within each site to generate the local propensity score and local weight for each individual. Third, we choose between global and local propensity score for each site based on covariate balance-related criterion, and use this chosen propensity score in each site to obtain the proposed weight for each individual. Fourth, we estimate the overall hazard ratio based on the proposed weight. All the above steps require only summary-level data to be transferred among sites, which would help protect individual privacy.

### Global and local propensity score

In the setting of distributed data with *K* sites, the propensity score can be estimated globally using data from the entire population or locally within each site.

Taking logistic regression models as an example, global propensity score is estimated by fitting logistic regression models to the overall sample:

2.1 $$\mathrm{logit}\left[e\left({\varvec{X}},\boldsymbol{\alpha }\right)\right]={{\delta }_{0,g}+\boldsymbol{\alpha }}_{g}^{\boldsymbol{^{\prime}}}{\varvec{X}}$$

Since we assume that data at the individual level cannot be shared among sites, data from the full sample cannot be directly used to fit model, and only summary-level statistics can be obtained from each site. The global propensity score can be obtained by distributed logistic regression. Let $e({\varvec{X}},{\boldsymbol{\alpha }}_{g})=P(A=1|{\varvec{X}}),$ and the logistic loss is

2.2 $${M}^{logis}\left(A,{\varvec{X}},{\boldsymbol{\alpha }}_{g}\right)=-\{A\mathrm{log}\left[e\left({\varvec{X}},{\boldsymbol{\alpha }}_{g}\right)\right]+(1-A)\mathrm{log}\left[1-e\left({\varvec{X}},{\boldsymbol{\alpha }}_{g}\right)\right]\}$$

The distributed Newton–Raphson method [15, 16] is used to obtain the empirical loss minimizer $\widehat{\alpha}\ {:=}\ arg\,min_{\alpha} \sum\nolimits_{i,k} M^{logis} \left(A_{i,k}, \boldsymbol{X}_{i,k}, \boldsymbol{\alpha}\right)$ through iterations:

2.3 $${\boldsymbol{\alpha }}^{(t+1)}={\boldsymbol{\alpha }}^{(t)}-{[{H}_{n}^{logis}\left({\boldsymbol{\alpha }}^{(t)}\right)]}^{-1}{G}_{n}^{logis}\left({\boldsymbol{\alpha }}^{(t)}\right) t=\mathrm{1,2},\dots$$

where ${G}_{n}^{logis}({\boldsymbol{\alpha }}^{(t)})=\frac{1}{n}{\sum }_{k=1}^{K}{\sum }_{i\in {\Omega }_{k}}{\nabla }_{\boldsymbol{\alpha }}{M}^{logis}\left({A}_{i},{{\varvec{X}}}_{i},{\boldsymbol{\alpha }}^{(t)}\right)$ is the global gradient and ${H}_{n}^{logis}({\boldsymbol{\alpha }}^{(t)})=\frac{1}{n}{\sum }_{k=1}^{K}{\sum }_{i\in {\Omega }_{k}}{\nabla }_{\alpha }^{2}{M}^{logis}\left({A}_{i},{{\varvec{X}}}_{i},{\boldsymbol{\alpha }}^{(t)}\right)$is the global Hessian matrix [15]. The iteration process is as follows:

1. Initialize ${\boldsymbol{\alpha }}^{(0)}={argmin}_{\boldsymbol{\alpha }}{\sum }_{i\in {\Omega }_{1}}{M}^{logis}\left({A}_{i},{{\varvec{X}}}_{i},\boldsymbol{\alpha }\right)$ based on data from the analysis center (e.g. site 1), and set $t=0$.
2. Repeat the following steps until $t$ meets the max iteration times or $\Vert {G}_{n}^{logis}\left(\boldsymbol{\alpha }\right)\Vert \le$ pre-specified threshold.
  - Transfer ${\boldsymbol{\alpha }}^{(t)}$ to each site to compute the local gradient ${G}_{{n}_{k}}^{logis}({\boldsymbol{\alpha }}^{(t)})$ and the local Hessian matrix ${H}_{{n}_{k}}^{logis}\left({\boldsymbol{\alpha }}^{\left(t\right)}\right)$, and transfer the local gradient and local Hessian matrix to the analysis center.
  - Calculate the global gradient ${G}_{n}^{logis}\left({\boldsymbol{\alpha }}^{(t)}\right)=\frac{1}{K}{\sum }_{k=1}^{K}{G}_{{n}_{k}}^{logis}({\boldsymbol{\alpha }}^{(t)})$ and the global Hessian matrix ${H}_{n}^{logis}\left({\boldsymbol{\alpha }}^{\left(t\right)}\right)=\frac{1}{K}{\sum }_{k=1}^{K}{H}_{{n}_{k}}^{logis}({\boldsymbol{\alpha }}^{(t)})$ in the analysis center.
  - Update ${\boldsymbol{\alpha }}^{(t)}$ in the analysis center as ${\boldsymbol{\alpha }}^{(t+1)}={\boldsymbol{\alpha }}^{(t)}-{[{H}_{n}^{logis}\left({\boldsymbol{\alpha }}^{(t)}\right)]}^{-1}{G}_{n}^{logis}\left({\boldsymbol{\alpha }}^{(t)}\right)$.

Then we could obtain the global propensity score ${\widehat{e}}_{g}=$ $e\left({\varvec{X}},{\widehat{\boldsymbol{\alpha }}}_{g}\right)$ based on the estimated parameter ${\widehat{\boldsymbol{\alpha }}}_{g}$ from iterations. It is worth noting that each site computes its own gradient and Hessian matrix, which are subsequently summarized to update the parameters. As a result, any site can be chosen as the analysis center. It is generally recommended to consider the hardware capabilities and computational power of each site when determining the analysis center.

An alternative approach is to estimate the local propensity score within each site:

2.4 $$\mathrm{logit}\left[e\left({\varvec{X}},\boldsymbol{\alpha }\right)\right]={{\delta }_{k}+\boldsymbol{\alpha }}_{l,k}^{\boldsymbol{^{\prime}}}{\varvec{X}}\boldsymbol{ }\boldsymbol{ }\boldsymbol{ } k=1, \dots ,K$$

We fit the model at each site using the observations from that site and obtain the local propensity score ${\widehat{e}}_{l}=$ $e\left({\varvec{X}},{\widehat{\boldsymbol{\alpha }}}_{l,k}\right)$ based on the estimated parameter ${\widehat{\boldsymbol{\alpha }}}_{l,k}$ from each site.

### Proposed propensity score

Motivated by Dong et al. [13] we propose a balancing propensity score to estimate the overall hazard ratio in distributed data to improve the estimation efficiency. The proposed method is to choose between the global and local propensity score by optimizing the overall confounder balance for propensity score weighting.

2.5 $${\widehat{\mathrm M}}_{\mathrm p}=\frac1{\mathrm n}\left[\sum\nolimits_{{\mathrm A}_{\mathrm i}=1}\frac1{{\widehat{\mathrm e}}_{\mathrm i}}{\mathrm x}_{\mathrm{ip}}-\sum\nolimits_{{\mathrm A}_{\mathrm i}=0}\frac1{1-{\widehat{\mathrm e}}_{\mathrm i}}{\mathrm x}_{\mathrm{ip}}\right]/{\widehat{\mathrm\sigma}}_{\mathrm p}$$

where ${\widehat{e}}_{i}$ is the estimated propensity score, ${x}_{ip}$ is the value of the *p*th measured confounder ${X}_{p}$ for individual $i$; ${\widehat{\sigma }}_{p}$ is the standard deviations of ${X}_{p}$ for overall population. ${\widehat{M}}_{p}$ accounts for balancing of confounder ${X}_{p}$ in the overall sample.

Notably, ${\widehat{M}}_{p}$ could not be directly estimated in distributed data and needs file transfer between sites.

${\widehat{M}}_{p}$ could be rewritten as:

2.6 $${\widehat{M}}_{p}=\frac{\frac{1}{n}\left[\sum_{k=1}^{K}\sum_{{A}_{i}=1,{G}_{i}=k}\frac{1}{{\widehat{e}}_{i}}{x}_{ip}-\sum_{k=1}^{K}\sum_{{A}_{i}=0,{G}_{i}=k}\frac{1}{1-{\widehat{e}}_{i}}{x}_{ip}\right]}{{\widehat{\sigma }}_{p}}$$

$${\widehat{\sigma }}_{p}=\frac{\left[\sum_{k=1}^{K}\sum_{{G}_{i}=k}{x}_{ip}^{2}-\frac{{\left(\sum_{k=1}^{K}\sum_{{G}_{i}=k}{x}_{ip}\right)}^{2}}{n}\right]}{n-1}$$

To obtain ${\widehat{M}}_{p}$, each site should transfer the following items to the analysis center:

1. $$\sum_{{A}_{i}=1,{G}_{i}=k}\frac{1}{{\widehat{e}}_{i}}{x}_{ip}$$ and $$\sum_{{A}_{i}=0,{G}_{i}=k}\frac{1}{1-{\widehat{e}}_{i}}{x}_{ip}$$.
2. $$\sum_{{G}_{i}=k}{x}_{ip}$$ and $$\sum_{{G}_{i}=k}{x}_{ip}^{2}$$.

${\widehat{M}}_{p}$ could then be calculated in the analysis center using these transferred values from each site based on (2.6). The objective function is the sum of the squares of ${\widehat{M}}_{p}.$

2.7 $$F=\sum_{p=1}^{P}{({\widehat{M}}_{p})}^{2}$$

We choose between global and local propensity scores for each site to minimize the objective function $F$.

### Stochastic search algorithm to estimate the proposed propensity score

Dong and others proposed a stochastic search algorithm to find the minimized objective function *F*in (2.7) [13]. For each site $k=1,\dots ,K$, let ${S}_{k}=1$ if individuals in site $k$ are weighted based on the estimated global propensity score, and ${S}_{k}=2$ if individuals in site $k$ are weighted based on the estimated local propensity score.

The search process is as follows:

1. Initially, let all sites use the global propensity score and ${S}_{k}=1$ for $k=1,\dots ,K$. The analysis center calculates the initial value ${F}_{int}$ for the objective function *F* using information transferred from each site. Let the minimum value ${F}_{min}={F}_{int}$, and let ${S}_{k,min}={S}_{k}=1$ for $k=1,\dots ,K$.
2. Repeat the following steps until the number of repeats is no smaller than ${L}_{1}$ or ${F}_{min}$ does not change over ${L}_{2}$ repeats. The values of ${L}_{1}$ and ${L}_{2}$ are pre-specified.
3. Randomly permutate all the sites $\{$ 1 $,2,$…$,$ K} and get a new random ordering of the *K* sites, {${A}_{1}, {A}_{2},\dots , {A}_{K}$}.
4. Following the order {${A}_{1}, {A}_{2},\dots , {A}_{K}$} in step (a), for each site, choose the global or local propensity score that gives a smaller value of objective function *F* while fixing the propensity score chosen for other $K-1$ sites each time. If site $k$ chooses the global propensity score, then ${S}_{k}=1$; if site $k$ chooses the local propensity score, then ${S}_{k}=2$.
5. After all sites have selected the global or local propensity score, calculate ${F}_{rep}$ for this repeat in the analysis center.
6. If ${F}_{rep}$ in step (c) is smaller than ${F}_{min}$, then update ${F}_{min}={F}_{rep}$ and ${S}_{k,min}={S}_{k}$; if ${F}_{rep}\ge {F}_{min}$, then keep ${F}_{min}$ and ${S}_{k,min}$ unchange.

For each site $k=1,\dots ,K$, if ${S}_{k,min}=1$ then the proposed propensity score for site $k$ is equal to the global propensity score; otherwise the proposed propensity score is equal to the local propensity score estimated within that site, i.e.,

$${\widehat{e}}_{p}={\widehat{e}}_{g} for site k, if {S}_{k,min}=1$$

$${\widehat{e}}_{p}={\widehat{e}}_{l} for site k, if {S}_{k,min}=2$$

### Estimation of overall hazard ratio with distributed survival data based on proposed propensity score

Based on the proposed propensity score ${\widehat{e}}_{i}={\widehat{e}}_{p,i}$, the inverse probability weight for individual $i$ is

$${\widehat{w}}_{i}=\frac{{A}_{i}}{{\widehat{e}}_{i}}+\frac{{1-A}_{i}}{1-{\widehat{e}}_{i}}$$

Then we could estimate the log hazard ratio $\widehat{\theta }$ by solving Eq. (1). In order to obtain $\widehat{\theta }$ in distributed data, Eq. (1) can be rewritten as

3.1 $$\sum_{k=1}^{K}\sum_{j=1}^{d(k)}\sum_{i\epsilon {D}_{k,j}(k)}{\widehat{w}}_{i}{A}_{i}-\sum_{k=1}^{K}\sum_{j=1}^{d\left(k\right)}\sum_{i\epsilon {D}_{k,j}\left(k\right)}{\widehat{w}}_{i}\frac{\sum_{{k}{\prime}=1}^{K}\sum_{l\epsilon {R}_{k,j}\left({k}{\prime}\right)}{\widehat{w}}_{l}\mathrm{exp}\left(\theta {A}_{l}\right){A}_{l}}{\sum_{{k}{\prime}=1}^{K}\sum_{l\epsilon {R}_{k,j}\left({k}{\prime}\right)}{\widehat{w}}_{l}\mathrm{exp}\left({\theta A}_{l}\right)}=0$$

$\sum_{l\epsilon {R}_{k,j}\left({k}{\prime}\right)}{\widehat{w}}_{l}\mathrm{exp}\left(\theta {A}_{l}\right){A}_{l}$ and $\sum_{l\epsilon {R}_{k,j}\left({k}{\prime}\right)}\widehat{{w}_{l}}\mathrm{exp}\left({\theta A}_{l}\right)$ in the score Eq. (3.1) can be expressed as

$$\sum_{l\epsilon {R}_{k,j}\left({k}{\prime}\right)}{\widehat{w}}_{l}\mathrm{exp}\left({\theta A}_{l}\right){A}_{l}=\mathrm{exp}\left(\theta \right)\sum_{l\epsilon {R}_{k,j}\left({k}{\prime}\right), {A}_{l}=1}{\widehat{w}}_{l}$$

$$\sum_{l\epsilon {R}_{k,j}\left({k}{\prime}\right)}{\widehat{w}}_{l}\mathrm{exp}\left({\theta A}_{l}\right)=\mathrm{exp}\left(\theta \right)\sum_{l\epsilon {R}_{k,j}\left({k}{\prime}\right), {A}_{l}=1}{\widehat{w}}_{l}+\sum_{l\epsilon {R}_{k,j}\left({k}{\prime}\right), {A}_{l}=0}{\widehat{w}}_{l}$$

Then the Eq. (3.1) can be further rewritten as

3.2 $$\sum_{k=1}^{K}\sum_{j=1}^{d(k)}\sum_{i\epsilon {D}_{k,j}\left(k\right)}{\widehat{w}}_{i}{A}_{i}-\sum_{k=1}^{K}\sum_{j=1}^{d\left(k\right)}\sum_{i\epsilon {D}_{k,j}\left(k\right)}{\widehat{w}}_{i}\frac{\mathrm{exp}\left(\theta \right)\sum_{{k}{\prime}=1}^{K}\sum_{l\epsilon {R}_{k,j}\left({k}{\prime}\right), {A}_{l}=1}{\widehat{w}}_{l}}{\mathrm{exp}\left(\theta \right)\sum_{{k}{\prime}=1}^{K}\sum_{l\epsilon {R}_{k,j}\left({k}{\prime}\right), {A}_{l}=1}{\widehat{w}}_{l}+\sum_{{k}{\prime}=1}^{K}\sum_{l\epsilon {R}_{k,j}\left({k}{\prime}\right), {A}_{l}=0}{\widehat{w}}_{l}}=0$$

To solve (3.2), we need to know: 

(u1)

$$\sum_{k=1}^{K}\sum_{j=1}^{d(k)}\sum_{i\epsilon {D}_{k,j}\left(k\right)}{\widehat{w}}_{i}{A}_{i}$$

(u2)

$$\sum_{k=1}^{K}\sum_{j=1}^{d\left(k\right)}\sum_{i\epsilon {D}_{k,j}\left(k\right)}{\widehat{w}}_{i}\frac{\mathrm{exp}\left(\theta \right)\sum_{{k}{\prime}=1}^{K}\sum_{l\epsilon {R}_{k,j}\left({k}{\prime}\right), {A}_{l}=1}{\widehat{w}}_{l}}{\mathrm{exp}\left(\theta \right)\sum_{{k}{\prime}=1}^{K}\sum_{l\epsilon {R}_{k,j}\left({k}{\prime}\right), {A}_{l}=1}{\widehat{w}}_{l}+\sum_{{k}{\prime}=1}^{K}\sum_{l\epsilon {R}_{k,j}\left({k}{\prime}\right), {A}_{l}=0}{\widehat{w}}_{l}}$$

Particularly in (u2), $\frac{\mathrm{exp}\left(\theta \right)\sum_{{k}{\prime}=1}^{K}\sum_{l\epsilon {R}_{k,j}\left({k}{\prime}\right), {A}_{l}=1}{\widehat{w}}_{l}}{\mathrm{exp}\left(\theta \right)\sum_{{k}{\prime}=1}^{K}\sum_{l\epsilon {R}_{k,j}\left({k}{\prime}\right), {A}_{l}=1}{\widehat{w}}_{l}+\sum_{{k}{\prime}=1}^{K}\sum_{l\epsilon {R}_{k,j}\left({k}{\prime}\right), {A}_{l}=0}{\widehat{w}}_{l}}$ should be calculated for all $d$ distinct observed event times across all sites. Therefore, each site needs to know the $d$ distinct observed event times, which requires each site to first send $d(k)$ observed event times in that site to the analysis center. Then the analysis center needs to summarize the event times from each site and send back all the $d$ distinct event times to each site. With information on $d$ distinct event times, each site $k$ could then calculate $\sum_{l\epsilon {R}_{j}(k), {A}_{l}=1}{\widehat{w}}_{l}$ and $\sum_{l\epsilon {R}_{j}(k), {A}_{l}=0}{\widehat{w}}_{l}$, and sends the results to the analysis center to sum up.

Detailed procedures to obtain the estimated log hazard ratio $\widehat{\theta }$ in distributed data:

- 1. Data transfer from each site *k* to the analysis center: each site transmits $d(k)$ distinct observed event times for site *k*,${\mathrm{T}}_{k,1}^{D},{\mathrm{T}}_{k,2}^{D},\dots ,{\mathrm{T}}_{k,d(k)}^{D}$, to the analysis center.
- 2. Data transfer from analysis center to each site: The analysis center summarizes the distinct observed event times across all sites, and transmits all $d$ event times, ${\mathrm{T}}_{1}^{D},{\mathrm{T}}_{2}^{D},\dots ,{\mathrm{T}}_{d}^{D}$, to each site.
- 3. Calculation in each site and data transfer from each site to the analysis center: Each site $k$ calculates $\sum_{l\epsilon {R}_{j}(k), {A}_{l}=1}{\widehat{w}}_{l}$ and $\sum_{l\epsilon {R}_{j}(k), {A}_{l}=0}{\widehat{w}}_{l}$ for $d$ distinct observed event times, and transmits the calculation result to the analysis center.
- 4. Data transfer from the analysis center to each site: Analysis center summarizes $\sum_{k=1}^{K}\sum_{l\epsilon {R}_{j}(k), {A}_{l}=1}{\widehat{w}}_{l}$ and $\sum_{k=1}^{K}\sum_{l\epsilon {R}_{j}(k), {A}_{l}=0}{\widehat{w}}_{l}$ for $d$ distinct observed event times, and transmits the summarized result to each site.
- 5. Data transfer from each site to the analysis center: For $d(k)$ distinct observed event times within each site, each site generates a summary-level table with 4 columns and $d(k)$ rows. The four columns are (i) $\sum_{i\epsilon {D}_{k,j}(k)}{\widehat{w}}_{i}{A}_{i}$, (ii) $\sum_{i\epsilon {D}_{k,j}(k)}{\widehat{w}}_{i}$, (iii) $\sum_{{k}{\prime}=1}^{K}\sum_{l\epsilon {R}_{k,j}\left({k}{\prime}\right), {A}_{l}=1}{\widehat{w}}_{l}$ for the $d(k)$ distinct observed event times ${T}_{k,j}^{D}$ in site *k*, (iv) $\sum_{{k}{\prime}=1}^{K}\sum_{l\epsilon {R}_{k,j}\left({k}{\prime}\right), {A}_{l}=0}{\widehat{w}}_{l}$ for the $d(k)$ distinct observed event times ${T}_{k,j}^{D}$ in site *k*. Each site transmits the 4-column table to the analysis center. An example of the 4-column summary table is presented in Table S1.
  In particular, for all $d$ distinct observed event times across all sites, $\sum_{{k}{\prime}=1}^{K}\sum_{l\epsilon {R}_{k,j}\left({k}{\prime}\right), {A}_{l}=1}{\widehat{w}}_{l}$ and $\sum_{{k}{\prime}=1}^{K}\sum_{l\epsilon {R}_{k,j}\left({k}{\prime}\right), {A}_{l}=0}{\widehat{w}}_{l}$ has been calculated and transmitted to each site in step 4. Therefore, for $d(k)$ distinct event times observed in site* k*, columns (iii) and (iv) can be directly obtained from file transfer in step 4.
- 6. The analysis center solves equation $(3.1)$ based on file transfer in step 5, and obtains the estimated log hazard ratio $\widehat{\uptheta }$.

## Simulations

### Simulation design

To examine the performance of the proposed method, we performed two sets of simulations. The first simulation was to compare the performance of our proposed method with the global propensity score for the entire population or local propensity score estimated within each site in distributed data with *K* sites. The second simulation was to compare our proposed method to the results obtained from the corresponding pooled individual-level data.

Assumed there were four covariates ${X}_{1}$~${X}_{4}$ and considered two scenarios:

- (a) Covariates and the treatment assignment in each site were homogenous: ${X}_{1} \sim \mathrm{Normal}(\mathrm{0,1})$, ${X}_{2} \sim \mathrm{Uniform}(\mathrm{0,1})$, ${X}_{3} \sim \mathrm{Normal}(\mathrm{0,1})$, ${X}_{4} \sim \mathrm{Bernoulli}(0.4)$. The treatment indicator $A$ was generated from the Bernoulli distribution according to the following propensity score model:

$$\mathrm{logit}\left[e\left({\varvec{X}},\boldsymbol{\alpha }\right)\right]={\delta }_{0}+{\alpha }_{1}{X}_{1}+{\alpha }_{2}{X}_{2}+{\alpha }_{3}{X}_{3}+{\alpha }_{4}{X}_{4}+{\alpha }_{5}{X}_{1}^{2}+{\alpha }_{6}{X}_{1}{X}_{4}$$

where $$\boldsymbol{\alpha }=\left({\alpha }_{1}, {\alpha }_{2},{\alpha }_{3},{\alpha }_{4},{\alpha }_{5},{\alpha }_{6}\right)=(-1.5, -0.5, 0.5, -0.5, 0.5, 1.5)$$ and $${\delta }_{0}=0.$$

(b) Covariates and the treatment assignment in each site were heterogeneous: ${X}_{1} \sim \mathrm{Normal}\left({\mu }_{k},1\right)$, if $G=k$, where ${\mu }_{k}=3-3\times \frac{\left(k-1\right)}{K-1}$, ${X}_{2} \sim \mathrm{Uniform}(\mathrm{0,1})$, ${X}_{3} \sim \mathrm{Normal}(\mathrm{0,1})$, ${X}_{4} \sim \mathrm{Bernoulli}(0.4)$. The treatment indicator $A$ was generated from the Bernoulli distribution according to the following propensity score model:

$$\mathrm{logit}\left[e\left({\varvec{X}},\boldsymbol{\alpha }\right)\right]=\sum_{k=1}^{K}{\delta }_{k}1\{G=k\}+{\alpha }_{1}{X}_{1}+{\alpha }_{2}{X}_{2}+{\alpha }_{3}{X}_{3}+{\alpha }_{4}{X}_{4}+{\alpha }_{5}{X}_{1}^{2}+{\alpha }_{6}{X}_{1}{X}_{5}$$

where $$\boldsymbol{\alpha }=\left({\alpha }_{1}, {\alpha }_{2},{\alpha }_{3},{\alpha }_{4},{\alpha }_{5},{\alpha }_{6}\right)=\left(-1.5, -0.5, 0.5, -0.5, 0.5, 1.5\right)$$ and $${\delta }_{k}=-1+2\times \frac{\left(k-1\right)}{K-1}$$.

Under each scenario, we also simulated the case where the treatment assignment model only included linear terms. Under homogenous scenario, the treatment indicator $A$ was generated from

$$\mathrm{logit}\left[e\left({\varvec{X}},\boldsymbol{\alpha }\right)\right]={\delta }_{0}+{\alpha }_{1}{X}_{1}+{\alpha }_{2}{X}_{2}+{\alpha }_{3}{X}_{3}+{\alpha }_{4}{X}_{4}$$

where $$\boldsymbol{\alpha }=\left({\alpha }_{1}, {\alpha }_{2},{\alpha }_{3},{\alpha }_{4}\right)=\left(-1.5, -0.5, 0.5, -0.5\right) \mathrm{and }{\delta }_{0}=0.$$

Under heterogeneous scenario, the treatment indicator $A$ was generated from

$$\mathrm{logit}\left[e\left({\varvec{X}},\boldsymbol{\alpha }\right)\right]=\sum_{k=1}^{K}{\delta }_{k}1\{G=k\}+{\alpha }_{1}{X}_{1}+{\alpha }_{2}{X}_{2}+{\alpha }_{3}{X}_{3}+{\alpha }_{4}{X}_{4}$$

where $$\boldsymbol{\alpha }=\left({\alpha }_{1}, {\alpha }_{2},{\alpha }_{3},{\alpha }_{4}\right)=\left(-1.5, -0.5, 0.5, -0.5\right) \mathrm{and }{\delta }_{k}=-1+2\times \frac{\left(k-1\right)}{K-1}.$$

For survival outcome, we defined $L=\mathrm{log}\left(1\right)A+\mathrm{log}\left(2\right){X}_{1}+\mathrm{log}\left(1.5\right){X}_{2}+\mathrm{log}\left(0.5\right){X}_{3}+\mathrm{log}\left(5\right){X}_{4}$, we generated ${T}^{*}$ from a Weibull distribution with a shape parameter of 2 and a scale parameter of ${0.5\mathrm{exp}(L)}^{-0.5}$. For censoring, we generated *C* from an exponential distribution with a rate parameter of $\mathrm{exp}(0.5)$. $T=\mathrm{min}\left({T}^{*},C\right)$ and $\delta =I({T}^{*}\le C)$.

In the stochastic search process, ${L}_{1}$ was set to be 500, and ${L}_{2}$ was set to be 20. We considered $K=5, 10, 20$ and ${n}_{k}=500, 1000, 2000$ to evaluate the impact of different numbers of sites and different sample sizes in each site on performance. We reported following performance measures: absolute bias, root mean squared error (RMSE), and ratio of RMSE of different methods against the proposed method (r-RMSE). We also presented the measure of coverage probability; however, due to constraints regarding computational costs, we only provided results for 5 sites. We compared three methods to generate weight for individual when estimating the overall hazard ratio: global weight (weight generated based on global propensity score ${\widehat{e}}_{g}$ for the entire population), local weight (weight generated based on local propensity score ${\widehat{e}}_{l}$ estimated within each site), and proposed weight (weight generated based on our proposed propensity score ${\widehat{e}}_{p}$). The statistical performance was evaluated based on 500 simulated datasets.

## Simulation results

When the covariates and the treatment assignment in each site were homogenous, the absolute bias was small for all the methods, i.e., weighted using global, local, and proposed propensity score. Compared with global weight and local weight, our proposed weight had a smaller RMSE, regardless of the number of sites and sample size in each site. The ratio of RMSE of the global or local weight to our proposed weight (r-RMSE) was up to 1.578 (Table 1).

**Table 1:** Comparisons of proposed weight, global weight and local weight to estimate the overall hazard ratio in the simulations, with homogenous design and treatment assignment generated with $X_1$~$X_4$ and $X_{1}^{2}$, $X_{1}X_{4}$

			$\boldsymbol K\boldsymbol=\mathbf5$			$\boldsymbol K\boldsymbol=\mathbf{10}$			$\boldsymbol K\boldsymbol=\mathbf{20}$	
		Global weight	Local weight	Proposed weight	Global weight	Local weight	Proposed weight	Global weight	Local weight	Proposed weight
${n}_{k}=500$	Bias	-0.018	-0.014	-0.017	-0.006	-0.012	-0.007	-0.002	-0.004	0.001
	RMSE	0.116	0.129	0.106	0.089	0.088	0.073	0.066	0.076	0.056
	r-RMSE	1.093	1.215	1.000 (Ref)	1.225	1.211	1.000 (Ref)	1.176	1.354	1.000 (Ref)
${n}_{k}=1000$	Bias	-0.006	-0.008	-0.006	-0.002	-0.003	-0.001	0.000	-0.004	0.001
	RMSE	0.089	0.100	0.077	0.066	0.068	0.058	0.122	0.093	0.087
	r-RMSE	1.160	1.303	1.000 (Ref)	1.140	1.175	1.000 (Ref)	1.401	1.068	1.000 (Ref)
${n}_{k}=2000$	Bias	-0.002	-0.003	-0.001	0.000	-0.004	-0.001	-0.002	-0.005	-0.002
	RMSE	0.066	0.065	0.061	0.122	0.082	0.077	0.050	0.045	0.040
	r-RMSE	1.087	1.071	1.000 (Ref)	1.578	1.060	1.000 (Ref)	1.249	1.124	1.000 (Ref)

*Bias* Absolute bias, *RMSE* Root mean squared error, *r-RMSE* Ratio of RMSE of global weight or local weight against proposed weight

In the heterogeneity setting, the absolute bias of our method was mostly somewhere between global and local weight, or close to that of global and local weight. Regarding RMSE, the RMSE of our proposed method remained the smallest, and the r-RMSE was up to 1.540 (Table 2). The results are similar when the treatment assignment was generated with ${\varvec{X}}=({X}_{1},{X}_{2},{X}_{3},{X}_{4})$ (Table 3, Table 4).

**Table 2:** Comparisons of proposed weight, global weight and local weight to estimate the overall hazard ratio in the simulations, with heterogeneous design and treatment assignment generated with $X_{1}$~$X_{4}$ and $X_{1}^{2}$, $X_{1}X_{4}$

			$\boldsymbol K\boldsymbol=\mathbf{5}$			$\boldsymbol K\boldsymbol=\mathbf{10}$			$\boldsymbol K\boldsymbol=\mathbf{20}$	
		Global weight	Local weight	Proposed weight	Global weight	Local weight	Proposed weight	Global weight	Local weight	Proposed weight
${n}_{k}=500$	Bias	0.045	0.041	0.033	0.026	0.023	0.015	0.025	0.019	0.009
	RMSE	0.129	0.125	0.110	0.082	0.097	0.065	0.069	0.070	0.049
	r-RMSE	1.176	1.140	1.000 (Ref)	1.255	1.484	1.000 (Ref)	1.400	1.421	1.000 (Ref)
${n}_{k}=1000$	Bias	0.039	0.024	0.022	0.027	0.018	0.012	0.031	0.020	0.012
	RMSE	0.096	0.092	0.076	0.083	0.079	0.067	0.105	0.084	0.072
	r-RMSE	1.269	1.216	1.000 (Ref)	1.247	1.187	1.000 (Ref)	1.458	1.166	1.000 (Ref)
${n}_{k}=2000$	Bias	0.031	0.014	0.011	0.037	0.021	0.017	0.021	0.005	0.002
	RMSE	0.093	0.094	0.077	0.196	0.175	0.161	0.042	0.049	0.032
	r-RMSE	1.210	1.223	1.000 (Ref)	1.215	1.084	1.000 (Ref)	1.320	1.540	1.000 (Ref)

*Bias* Absolute bias, *RMSE* Root mean squared error, *r-RMSE* Ratio of RMSE of global weight or local weight against proposed weight

**Table 3:** Comparisons of proposed weight, global weight and local weight to estimate the overall hazard ratio in the simulations, with homogenous design and treatment assignment generated with $X_{1}$~$X_{4}$

			$\boldsymbol K\boldsymbol=\mathbf{5}$			$\boldsymbol K\boldsymbol=\mathbf{10}$			$\boldsymbol K\boldsymbol=\mathbf{20}$	
		Global weight	Local weight	Proposed weight	Global weight	Local weight	Proposed weight	Global weight	Local weight	Proposed weight
${n}_{k}=500$	Bias	-0.009	-0.011	-0.014	0.003	0.000	-0.001	0.003	0.000	0.002
	RMSE	0.104	0.106	0.098	0.071	0.073	0.065	0.053	0.055	0.048
	r-RMSE	1.063	1.084	1.000 (Ref)	1.087	1.118	1.000 (Ref)	1.106	1.148	1.000 (Ref)
${n}_{k}=1000$	Bias	0.003	0.002	0.000	0.003	0.002	0.002	0.002	0.000	0.000
	RMSE	0.071	0.074	0.068	0.053	0.054	0.049	0.035	0.036	0.032
	r-RMSE	1.046	1.091	1.000 (Ref)	1.083	1.104	1.000 (Ref)	1.078	1.109	
${n}_{k}=2000$	Bias	0.003	0.002	0.002	0.002	0.001	0.001	-0.001	-0.001	-0.001
	RMSE	0.053	0.052	0.050	0.035	0.035	0.032	0.026	0.026	0.024
	r-RMSE	1.064	1.044	1.000 (Ref)	1.087	1.087	1.000 (Ref)	1.069	1.069	1.000 (Ref)

*Bias* Absolute bias, *RMSE* Root mean squared error, *r-RMSE* Ratio of RMSE of global weight or local weight against proposed weight

**Table 4:** Comparisons of proposed weight, global weight and local weight to estimate the overall hazard ratio in the simulations, heterogeneous design and treatment assignment generated with $X_{1}$~$X_{4}$

			$\boldsymbol K\boldsymbol=\mathbf{5}$			$\boldsymbol K\boldsymbol=\mathbf{10}$			$\boldsymbol K\boldsymbol=\mathbf{20}$	
		Global weight	Local weight	Proposed weight	Global weight	Local weight	Proposed weight	Global weight	Local weight	Proposed weight
${n}_{k}=500$	Bias	-0.098	-0.140	-0.119	-0.064	-0.098	-0.079	-0.035	-0.073	-0.049
	RMSE	0.287	0.254	0.239	0.208	0.226	0.188	0.171	0.178	0.139
	r-RMSE	1.200	1.062	1.000 (Ref)	1.106	1.201	1.000 (Ref)	1.232	1.283	1.000 (Ref)
${n}_{k}=1000$	Bias	-0.071	-0.073	-0.071	-0.040	-0.049	-0.046	-0.031	-0.028	-0.028
	RMSE	0.221	0.247	0.206	0.178	0.174	0.147	0.124	0.144	0.101
	r-RMSE	1.071	1.197	1.000 (Ref)	1.215	1.188	1.000 (Ref)	1.231	1.430	1.000 (Ref)
${n}_{k}=2000$	Bias	-0.040	-0.031	-0.038	-0.034	-0.017	-0.025	-0.033	-0.019	-0.019
	RMSE	0.210	0.199	0.170	0.128	0.142	0.112	0.106	0.100	0.078
	r-RMSE	1.235	1.170	1.000 (Ref)	1.142	1.267	1.000 (Ref)	1.351	1.274	1.000 (Ref)

*Bias* Absolute bias. *RMSE* Root mean squared error, *r-RMSE* Ratio of RMSE of global weight or local weight against proposed weight

Besides, we have computed the 95% coverage probability for 5 sites, and our proposed method achieved a coverage probability close to the nominal 95%, and was closer to the nominal 95% compared to the global and local method (Table 5).

**Table 5:** The coverage probability of different propensity score methods with the number of sites set to 5

		Setting 1	
	Global weight	Local weight	Proposed weight
${n}_{k}=500$	88.2	90.0	91.2
${n}_{k}=1000$	93.0	93.0	95.0
${n}_{k}=2000$	90.8	91.4	92.8
		Setting 2	
	Global weight	Local weight	Proposed weight
${n}_{k}=500$	88.6	91.0	92.8
${n}_{k}=1000$	89.0	91.0	93.2
${n}_{k}=2000$	87.2	90.2	94.4

Setting 1: homogenous design and treatment assignment generated with ${X}_{1}$~${X}_{4}$ and ${X}_{1}^{2}$, ${X}_{1}{X}_{4}$; setting 2: heterogeneous design and treatment assignment generated with ${X}_{1}$~${X}_{4}$ and ${X}_{1}^{2}$, ${X}_{1}{X}_{4}$

When comparing our proposed method to the results obtained from the corresponding pooled individual-level data analysis, as expected, our proposed method in distributed data and pooled individual-level data analysis yielded identical results under all scenarios (Table 6).

**Table 6:** Comparisons of the proposed method in distributed data and corresponding pooled individual-level data analysis

Distributed Data Analysis							
			Setting 1			Setting 2	
		Global weight	Local weight	Proposed weight	Global weight	Local weight	Proposed weight
$K=5$, ${n}_{k}=500$	Bias	-0.018	-0.014	-0.017	0.045	0.041	0.033
	RMSE	0.116	0.129	0.106	0.129	0.125	0.110
	r-RMSE	1.093	1.215	1.000 (Ref)	1.176	1.140	1.000 (Ref)
$K=5$, ${n}_{k}=1000$	Bias	-0.006	-0.008	-0.006	0.039	0.024	0.022
	RMSE	0.089	0.100	0.077	0.096	0.092	0.076
	r-RMSE	1.160	1.303	1.000 (Ref)	1.269	1.216	1.000 (Ref)
$K=5$, ${n}_{k}=2000$	Bias	-0.002	-0.003	-0.001	0.031	0.014	0.011
	RMSE	0.066	0.065	0.061	0.093	0.094	0.077
	r-RMSE	1.087	1.071	1.000 (Ref)	1.210	1.223	1.000 (Ref)
Pooled Individual-Level Data Analysis							
			Setting 1			Setting 2	
		Global weight	Local weight	Proposed weight	Global weight	Local weight	Proposed weight
$K=5$, ${n}_{k}=500$	Bias	-0.018	-0.014	-0.017	0.045	0.041	0.033
	RMSE	0.116	0.129	0.106	0.129	0.125	0.110
	r-RMSE	1.093	1.215	1.000 (Ref)	1.176	1.140	1.000 (Ref)
$K=5$, ${n}_{k}=1000$	Bias	-0.006	-0.008	-0.006	0.039	0.024	0.022
	RMSE	0.089	0.100	0.077	0.096	0.092	0.076
	r-RMSE	1.160	1.303	1.000 (Ref)	1.269	1.216	1.000 (Ref)
$K=5$, ${n}_{k}=2000$	Bias	-0.002	-0.003	-0.001	0.031	0.014	0.011
	RMSE	0.066	0.065	0.061	0.093	0.094	0.077
	r-RMSE	1.087	1.071	1.000 (Ref)	1.210	1.223	1.000 (Ref)

*Bias* absolute bias, *RMSE *root mean squared error, *r-RMSE* ratio of RMSE of global weight or local weight against proposed weight

Setting 1: homogenous design and treatment assignment generated with ${X}_{1}$~${X}_{4}$ and ${X}_{1}^{2}$, ${X}_{1}{X}_{4}$; setting 2: heterogeneous design and treatment assignment generated with ${X}_{1}$~${X}_{4}$ and ${X}_{1}^{2}$, ${X}_{1}{X}_{4}$

## Application

We apply the proposed method to real-world triple-negative breast cancer (TNBC) data from Surveillance, Epidemiology, and End Results (SEER) [17]. TNBC is an aggressive subtype of breast cancer, accounting for about 20% of all breast cancer cases [18]. It is known that radiation therapy can improve locoregional control in breast cancer patients and has a positive impact on the long-term survival of high-risk patients [19].

The dataset included 4120 patients aged 20–79 years diagnosed with TNBC in 2010 with complete information. The treatment variable was set to 1 if the patient received radiation therapy and 0 if not. The outcome of interest was the time to death during the follow-up of up to 71 months. Descriptive characteristics of patients according to radiation therapy were presented in Table S2. We estimated the hazard ratio and 95% confidence interval after adjusting for age, race, marital status, laterality, grade, the American Joint Committee on Cancer (AJCC) stage, surgery, distant metastasis, and chemotherapy in the propensity score model.

The patients were from five states: Connecticut (${n}_{1}=717$), Hawaii (${n}_{2}=274$), Iowa (${n}_{3}=723$), Kentucky (${n}_{4}=1176$), and Louisiana (${n}_{5}=1230$). Descriptive characteristics of patients according to five sites were presented in Table S3. We compared the proposed method with methods based on the global or local propensity score in the distributed survival data with 5 sites. We further compared the estimates from proposed methods in distributed data to estimates from the corresponding pooled individual-level data analyses.

The confidence intervals were calculated using the bootstrap method with 200 replications [11]. All $n$ individuals in $K$ sites were assigned ID of $\{\mathrm{1,2},\dots n\}$. In each bootstrap replication, the analysis center re-sampled with replacement from $\{\mathrm{1,2},\dots n\}$ and sent the re-sampled ID of the 200 replications to each site. Each site then prepared 200 bootstrap samples based on the instruction from the analysis center. The sample size of the resulting bootstrap samples for each site may differ from that site's original size.

Table 7 presented the estimated hazard ratio and their 95% confidence intervals. Results from the proposed methods and methods based on global or local propensity score indicated that radiation therapy had a positive impact on long-term survival in patients with TNBC. The proposed method was more likely to find a significant effect (hazard ratio, 0.679; 95% confidence interval, 0.585 to 0.789) compared to the global propensity score method (0.737; 0.653 to 0.832) and local propensity score method (0.709; 0.619 to 0.812). Besides, the proposed method and methods based on global or local propensity score produced hazard ratio estimates and 95% confidence intervals equivalent to those obtained from the corresponding pooled individual-level data analyses.

**Table 7:** Estimation of overall hazard ratio and the corresponding 95% confidence intervals using different propensity score estimation methods in distributed data and pooled individual-level data

Distributed Data Analysis		
Method	Hazard ratio	95% Confidence intervals
Global weight	0.737	0.653 to 0.832
Local weight	0.709	0.619 to 0.812
Proposed weight	0.679	0.585 to 0.789
Pooled Individual-Level Data Analysis		
Method	Hazard ratio	95% Confidence intervals
Global weight	0.735	0.651 to 0.831
Local weight	0.708	0.618 to 0.811
Proposed weight	0.669	0.575 to 0.777

## Discussion

We have proposed a covariate balance-related propensity score to create inverse probability weight to make inferences on the overall hazard ratio in multi-site distributed survival data. This proposed propensity score is produced based on covariate balance-related criterion in the entire population. The proposed propensity score is shown to perform better than the global propensity score estimated using data from the entire population or the local propensity score estimated within each site. Besides, the proposed method could be conducted without individual-level data transferred among sites and would yield identical results to the corresponding pooled individual-level data analysis.

The proposed method is developed based on distributed data with multiple sites. Since our proposed method in distributed data and pooled individual-level data analysis yield identical results, the proposed method can be extended to the general studies that data is distributed in multiple sites, but data communication among sites is not restricted. Therefore, in multi-site data, whether or not data transmission between sites is allowed, we recommend using our proposed approach and selecting between the global and local propensity score in each site to estimate the overall treatment effect with efficiency.

In our real-world data analysis, we calculated the 95% confidence intervals based on the global bootstrap method, which re-sampled from the entire population. In practice, researchers can also use the alternative local bootstrap method for simplicity [11]. Specifically, each site could generate its 200 or more bootstrap samples with replacement from the original sample in that site, which is the conventional bootstrap method within the site. We also applied the local bootstrap method to the real-world data, and the result was similar and presented in Table S4.

Our method is proposed based on the unstratified Cox model. Sometimes, if we assume the baseline hazard to vary by site, stratification on site is helpful and the stratified Cox model is used accordingly. In this case, the stratified Breslow-type weighted partial likelihood would be used instead of (1) in our study [11]. The main difference is that each site no longer needs to know the information of all $d$ distinct observed event times across all sites, but only needs to obtain its own summarized information of $d(k)$ distinct observed event times. Accordingly, the detailed steps 1 to 5 in calculating the overall hazard ratio in our study can be replaced by a simple step, i.e., to obtain the following information within each site: (i) $\sum_{i\epsilon {D}_{k,j}(k)}{\widehat{w}}_{i}{A}_{i}$, (ii) $\sum_{i\epsilon {D}_{k,j}(k)}{\widehat{w}}_{i}$, (iii) $\sum_{l\epsilon {R}_{k,j}\left({k}{\prime}\right), {A}_{l}=1}{\widehat{w}}_{l}$ for the $d(k)$ distinct observed event times ${T}_{k,j}^{D}$ in site *k*, (iv) $\sum_{l\epsilon {R}_{k,j}\left({k}{\prime}\right), {A}_{l}=0}{\widehat{w}}_{l}$ for the $d(k)$ distinct observed event times ${T}_{k,j}^{D}$ in site *k*. Under such circumstances, only one file transfer from each site to the analysis center is required after obtaining the proposed propensity score.

When conducting propensity score-based analysis, it is crucial to correctly identify the set of confounders and specify the propensity score model. We assume that all confounding variables are measurable and known in our study, and that there is no misclassification, missing data, or time-varying covariates. In future studies, it is possible to consider extending our method to situations where these assumptions are not satisfied or data with a large number of candidate covariates [20, 21].

## Conclusions

In this study, we proposed a covariate balance-related propensity score to estimate the overall hazard ratio, which only required summary-level information across sites to provide personal privacy protection. The proposed propensity score was estimated based on covariate balance-related criterion, and was shown to outperform the global propensity score estimated using data from the entire population or the local propensity score estimated within each site.

### Supplementary Information

**Additional file 1: Table S1. **An example of the 4-column summary table transferred from the site to the analysis center (10 rows are shown for illustration).**Table S2. **Descriptive characteristics of patients according to radiation therapy in real-world data analysis. Values are numbers (percentages) of individuals unless otherwise stated. **Table S3. **Descriptive characteristics of patients according to five sites in real-world data analysis. Values are numbers (percentages) of individuals unless otherwise stated. **Table S4. **Hazard ratios and 95% confidence intervals in real-world data analysis with local bootstrap.

## Data Availability

Publicly available datasets were analyzed in this study. These data can be found here: https://seer.cancer.gov/data-software/.
