# Supplementary material for: Covariate balance-related propensity score weighting in estimating overall hazard ratio with distributed survival data
Source: BMC Med Res Methodol. 2023 Oct 13;23:233. doi: 10.1186/s12874-023-02055-8 (PMC10576397; doi:10.1186/s12874-023-02055-8)
Supplement: Supplementary file 1 — Additional file 1: Table S1. An example of the 4-column summary table transferred from the site to the analysis center (10 rows are shown for illustration).Table S2. Descriptive characteristics of patients according to radiation therapy in real-world data analysis. Values are numbers (percentages) of individuals unless otherwise stated. Table S3. Descriptive characteristics of patients according to five sites in real-world data analysis. Values are numbers (percentages) of individuals unless otherwise stated. Table S4. Hazard ratios and 95% confidence intervals in real-world data analysis with local bootstrap. [file 12874_2023_2055_MOESM1_ESM.docx]

**Covariate balance-related propensity score weighting in estimating overall hazard ratio with distributed survival data**

**Table S1. An example of the 4-column summary table transferred from the site to the analysis center (10 rows are shown for illustration).**

| $\sum_{i\epsilon D_{k,j}(k)} \hat{w}_{i}A_{i}$ | $\sum_{i\epsilon D_{k,j}(k)} \hat{w}_{i}$ | $\sum_{k^{'}=1}^{K} \sum_{l\epsilon R_{k,j}\left( k^{'} \right), A_{l}=1} \hat{w}_{l}$ | $\sum_{k^{'}=1}^{K} \sum_{l\epsilon R_{k,j}\left( k^{'} \right), A_{l}=0} \hat{w}_{l}$ |
| --- | --- | --- | --- |
| 7.131 | 7.131 | 3556.772 | 3561.703 |
| 0.000 | 4.590 | 3526.701 | 3521.536 |
| 1.802 | 7.328 | 3498.754 | 3463.380 |
| 1.583 | 1.583 | 3471.102 | 3413.468 |
| 3.848 | 4.950 | 3443.763 | 3358.717 |
| 4.204 | 10.034 | 3422.369 | 3347.361 |
| 3.038 | 7.051 | 3399.798 | 3309.598 |
| 0.000 | 1.919 | 3371.213 | 3242.805 |
| 3.171 | 3.171 | 3348.169 | 3220.670 |
| 2.785 | 4.981 | 3328.069 | 3196.268 |

**Table S2. Descriptive characteristics of patients according to radiation therapy in real**-**world data analysis. Values are numbers (percentages) of individuals unless otherwise stated.**

|  | Treatment (N=2366) | Control (N=1754) | P value |
| --- | --- | --- | --- |
| Outcome |  |  | <0.001 |
| No | 2018 (85.3) | 1369 (78.1) |  |
| Yes | 348 (14.7) | 385 (21.9) |  |
| Age (mean (SD)) | 56.23 (11.49) | 55.13 (12.42) | 0.003 |
| Marital status |  |  | 0.074 |
| Unmarried | 921 (38.9) | 732 (41.7) |  |
| Married | 1445 (61.1) | 1022 (58.3) |  |
| Race |  |  | 0.011 |
| White | 1681 (71.0) | 1317 (75.1) |  |
| Black | 530 (22.4) | 348 (19.8) |  |
| Others | 155 (6.6) | 89 (5.1) |  |
| Lateral |  |  | 0.062 |
| Left | 1251 (52.9) | 875 (49.9) |  |
| Right | 1115 (47.1) | 879 (50.1) |  |
| Grade |  |  | 0.897 |
| Poorly | 377 (15.9) | 276 (15.7) |  |
| Others | 1989 (84.1) | 1478 (84.3) |  |
| Stage |  |  | 0.001 |
| Ⅰ/Ⅱ | 1864 (78.8) | 1458 (83.1) |  |
| Ⅲ/Ⅳ | 502 (21.2) | 296 (16.9) |  |
| Surgery |  |  | <0.001 |
| No | 38 (1.6) | 156 (8.9) |  |
| Yes | 2328 (98.4) | 1598 (91.1) |  |
| Distant metastasis | |  | <0.001 |
| No | 2217 (93.7) | 1583 (90.3) |  |
| Yes | 149 (6.3) | 171 (9.7) |  |
| Chemotherapy |  |  | <0.001 |
| No/unknown | 306 (12.9) | 431 (24.6) |  |
| Yes | 2060 (87.1) | 1323 (75.4) |  |

**Table S3. Descriptive characteristics of patients according to five sites in real**-**world data analysis. Values are numbers (percentages) of individuals unless otherwise stated.**

|  | Site 1 (N=717) | Site 2 (N=274) | Site 3 (N=723) | Site 4 (N=1176) | Site 5 (N=1230) |
| --- | --- | --- | --- | --- | --- |
| Treatment |  |  |  |  |  |
| No | 340 (47.4) | 95 (34.7) | 294 (40.7) | 502 (42.7) | 523 (42.5) |
| Yes | 377 (52.6) | 179 (65.3) | 429 (59.3) | 674 (57.3) | 707 (57.5) |
| Outcome |  |  |  |  |  |
| No | 613 (85.5) | 234 (85.4) | 597 (82.6) | 947 (80.5) | 996 (81.0) |
| Yes | 104 (14.5) | 40 (14.6) | 126 (17.4) | 229 (19.5) | 234 (19.0) |
| Age (mean (SD)) | 55.19 (12.51) | 57.61 (12.02) | 56.56 (12.25) | 55.73 (11.52) | 55.23 (11.63) |
| Marital status | |  |  |  |  |
| Unmarried | 285 (39.7) | 106 (38.7) | 232 (32.1) | 437 (37.2) | 593 (48.2) |
| Married | 432 (60.3) | 168 (61.3) | 491 (67.9) | 739 (62.8) | 637 (51.8) |
| Race |  |  |  |  |  |
| White | 592 (82.6) | 67 (24.5) | 695 (96.1) | 1038 (88.3) | 606 (49.3) |
| Black | 102 (14.2) | 11 (4.0) | 22 (3.0) | 131 (11.1) | 612 (49.8) |
| Others | 23 (3.2) | 196 (71.5) | 6 (0.8) | 7 (0.6) | 12 (1.0) |
| Lateral |  |  |  |  |  |
| Left | 382 (53.3) | 150 (54.7) | 378 (52.3) | 566 (48.1) | 650 (52.8) |
| Right | 335 (46.7) | 124 (45.3) | 345 (47.7) | 610 (51.9) | 580 (47.2) |
| Grade |  |  |  |  |  |
| Poorly | 107 (14.9) | 65 (23.7) | 75 (10.4) | 208 (17.7) | 198 (16.1) |
| Others | 610 (85.1) | 209 (76.3) | 648 (89.6) | 968 (82.3) | 1032 (83.9) |
| Stage |  |  |  |  |  |
| Ⅰ/Ⅱ | 594 (82.8) | 233 (85.0) | 581 (80.4) | 954 (81.1) | 960 (78.0) |
| Ⅲ/Ⅳ | 123 (17.2) | 41 (15.0) | 142 (19.6) | 222 (18.9) | 270 (22.0) |
| Surgery |  |  |  |  |  |
| No | 46 (6.4) | 8 (2.9) | 28 (3.9) | 46 (3.9) | 66 (5.4) |
| Yes | 671 (93.6) | 266 (97.1) | 695 (96.1) | 1130 (96.1) | 1164 (94.6) |
| Distant metastasis | |  |  |  |  |
| No | 673 (93.9) | 253 (92.3) | 665 (92.0) | 1089 (92.6) | 1120 (91.1) |
| Yes | 44 (6.1) | 21 (7.7) | 58 (8.0) | 87 (7.4) | 110 (8.9) |
| Chemotherapy | |  |  |  |  |
| No/unknown | 125 (17.4) | 62 (22.6) | 136 (18.8) | 219 (18.6) | 195 (15.9) |
| Yes | 592 (82.6) | 212 (77.4) | 587 (81.2) | 957 (81.4) | 1035 (84.1) |

**Table S4. Hazard ratios and 95% confidence intervals in real**-**world data analysis with local bootstrap.**

| Method | Hazard ratio | 95% Confidence intervals |
| --- | --- | --- |
| Global weight | 0.737 | 0.647 to 0.840 |
| Local weight | 0.709 | 0.622 to 0.807 |
| Proposed weight | 0.684 | 0.580 to 0.806 |
